# Protective effects of *Aloe vera* extract against doxorubicin-induced degeneration in ovarian follicles and stromal cells in mice

**DOI:** 10.1590/1414-431X2025e14402

**Published:** 2025-06-16

**Authors:** E.I.T. de Assis, A.N. Godinho, J.M.O. Freire, M.F. de Lima, J.J.N. Costa, A.L.P. Souza, A.P.O. do Monte, M.H.T. Matos, A.L.M. de Sousa, J.R.V. Silva, A.W.B. Silva

**Affiliations:** 1 Laboratório de Biotecnologia e Fisiologia da Reprodução, Universidade Federal do Ceará, Sobral, CE, Brasil; 2 Núcleo de Pesquisa em Experimentação Animal, Universidade Federal do Ceará, Sobral, CE, Brasil; 3 Núcleo de Biotecnologia Aplicada ao Desenvolvimento do Folículo Ovariano, Universidade Federal do Vale do São Francisco, Petrolina, PE, Brasil; 4 Embrapa Caprinos e Ovinos, Fazenda Três Lagoas, Sobral, CE, Brasil

**Keywords:** Aloe vera, Doxorubicin, Chemotherapy, Mice, Fertility

## Abstract

The present study aimed to evaluate the protective effects of *Aloe vera* on doxorubicin (DOX)-induced degeneration in ovarian follicles and stromal cells in mice. Mice (n=48) were randomly divided into six groups. The positive control group mice received pretreatment of N-acetylcysteine orally (*po*), followed by a single intraperitoneal (*ip*) dose of DOX after 1 h (NAC+DOX). The negative control group mice were pre-treated with saline (*po*) and administered a single DOX dose (*ip*) after 1 h (SAL+DOX). The other groups of mice were pre-treated with different concentrations (0.1, 1.0, or 10.0 mg/kg; *po*) of *Aloe vera* and then received a single dose of DOX (*ip*) after 1 h (AV0.1+DOX, AV1.0+DOX, and AV10.0+DOX). The control group received saline *po* and *ip* (SAL+SAL). *Aloe vera* was administered once daily for 3 consecutive days. On the fourth day, the ovaries were processed for histological analysis, immunohistochemistry, and real-time PCR (mRNA for superoxide dismutase (*SOD*), catalase (*CAT*), nuclear factor erythroid 2-related factor 2 (*NRF2*), and tumor necrosis factor-α *(TNF*-*α)*. Results showed that 0.1 and 1.0 mg/kg *Aloe vera* protected ovarian follicles and stromal density against DOX-induced degeneration. Furthermore, 0.1 and 1.0 mg/kg *Aloe vera* reduced TNF-α protein expression and increased *NRF2*, *SOD*, and *CAT* mRNA levels. In conclusion, 0.1 and 1.0 mg/kg *Aloe vera* had protective effects against DOX-induced degeneration in ovarian follicles and stromal cells in mice.

## Introduction

Cancer is a public health problem worldwide and it is expected that by 2030 there will be over 25 million new cases (1). Advances in chemotherapy have improved the prognosis of cancer patients, and the survival rate has increased for most cancers, including those affecting young women (2). Doxorubicin (DOX), a broad-spectrum antineoplastic drug, is effective in a variety of tumors, including sarcomas (3), but has deleterious effects on female fertility, even at low doses (4). DOX exerts anticancer activities by mainly inhibiting DNA replication and RNA transcription, inhibiting topoisomerase II activity, and inducing free radical production and peroxidation (5). The toxic effects of DOX in the ovary have been associated with reduced antioxidant capacity, increased production of reactive oxygen species (ROS), mitochondrial damage, and inflammatory response, leading to cell apoptosis (6).

The administration of natural substances before or during antineoplastic chemotherapy has emerged as a strategy to prevent ovarian damage and preserve fertility in cancer patients (7). Recently, Silva et al. (6) showed that gallic acid protects the mouse ovary against DOX-induced damage by improving glutathione reductase (GSH) concentrations, mitochondrial activity, and cell proliferation, inhibiting inflammation and apoptosis. Furthermore, it has been observed that rutin has the potential to protect ovarian follicles against cisplatin-induced toxicity through its antioxidant effects via PTEN/FOXO3a (8). Oxidative stress and inflammation induced by antineoplastic drugs have been associated to ovarian injury, and the use of natural compounds with antioxidant and anti-inflammatory properties have been highlighted (6,8).

*Aloe vera* is a drought-resistant stemless succulent plant of the lily family native to warm climates and has been used medicinally by different cultures. Recently, Atiba et al. (9) reported that *Aloe vera* extract has anti-inflammatory and antioxidant effects. *Aloe vera* extract contains a large amount of antioxidant substances (Table 1; 10-15) that are very efficient in modulating or inactivating ROS (16). Although many positive effects are attributed to polysaccharides, it is believed that biological activities result from the synergistic action of various compounds, rather than a single chemical substance. *Aloe vera* extract effectively inhibits inflammatory reactions by inhibiting interleukin (IL)-6 and IL-8, reducing leukocyte adhesion, increasing IL-10 levels, and decreasing tumor necrosis factor (TNF)-α levels (9). *In vitro* studies show that *Aloe vera* extract improves extracellular matrix distribution and increases mRNA expression for peroxiredoxin 6 (*PRDX6)* in bovine ovarian tissue cultured *in vitro* (17). Moreover, catalase (CAT) is an important enzyme that scavenges H_2_O_2_ and indirectly inhibits lipid peroxidation and membrane damage. The expression of antioxidant enzymes is positively regulated by transcription factor nuclear factor erythroid 2-related factor 2 (NRF-2) (18). Recently, the therapeutic benefits of *Aloe vera* as adjunctive therapy have been observed in the treatment of polycystic ovary syndrome (PCOS) in female mice (19). Thus, it is hypothesized that the bioactive compounds present in *Aloe vera* exert antioxidant and anti-inflammatory effects, which may protect the ovaries from the harmful effects of DOX, contributing to the preservation of ovarian health and mitigating the adverse side effects of chemotherapy.

**Table 1:** Chemical composition of *Aloe vera* leaf extract.

Class	Components	Effects	Reference
Polyphenols	Benzoic acid, p-toluic acid, p-coumaric acid, p-salicylic acid, protocatechuic acid, hydroxyphenylacetic acid, ferulic acid, aloe emodin, and vanillic acid	Antioxidant	10
Polysaccharides	Galactans, pectic substances, and polysaccharides containing glucuronic acid, fucose, arabinose, xylose, and rhamnose	Antibacterial, anti-inflammatory, and antiviral	11
Anthraquinone	Aloin (A and B), barbaloin, isobarbaloin, emodin, aloe-emodin, rhein, and chrysophanol	Antibacterial, antiviral, anti-inflammatory, antifungal, anticancer, and antioxidant	12
Polysaccharides	Glucose, mannose, galactose, and arabinose	Anti-genotoxic and antitumor	13
Carbohydrate	Acemannan	Immunomodulation/radioprotection	14
Minerals	Calcium, chromium, iron, magnesium, manganese, potassium, sodium, and zinc	Maintenance of physiological processes	15

The present study aimed to evaluate whether *Aloe vera* extract helps preserve follicular morphology and development, ovarian stromal cell density, and collagen fiber in the extracellular matrix by regulating mRNA expression of *SOD*, *CAT*, *TNF*-α, and *NRF2*, as well as the expression of TNF-α protein in mice treated with DOX.

## Material and Methods

### *Aloe vera* extract

The *Aloe vera* extract was obtained from the leaves of the plant at an intermediate stage of maturity. For this, the plant was cultivated in a region with temperatures ranging from 24°C to 36°C, a semi-arid tropical climate. Leaves were collected using properly sanitized slides and transported to the laboratory. In the laboratory, the leaves were washed with distilled water and their surface was removed to expose the liquid and clear gel (17). For lyophilization, the gel was homogenized and transferred to a sterile 15-mL falcon tube and stored for 24 h at -80°C, then the tubes were kept in the ScanVac CoolSafe Touch Superior lyophilizer (LaboGene A/S, Denmark) for 4 days (20). The lyophilized extract obtained was then diluted in saline solution for administration to the animals.

### Chemicals

DOX (2 mg/mL injectable solution) was purchased from Libbs Fauldoxo^®^ (Brazil), while the other chemicals used in this study were from Sigma Chemical Co. (USA).

### Animals and estrous cycle evaluation

This study was performed in accordance with the guidelines from the Ethics Committee on the Use of Animals (CEUA) of the Federal University of Ceará (approved under protocol No. 08/20). Adult *Swiss* female mice (*Mus musculus*, 18 g and/or 2 months old) were kept in boxes, at an average temperature of 22±2°C, with free access to filtered water and food in 12-h light-dark cycles. During a period of 15 days, the estrous cycle was evaluated once a day, between 9:00 and 10:00 a.m., as established by Marcondes et al. (21). According to the cell type, the estrus cycle stage was classified as proestrus, estrus, metestrus, or diestrus. Only mice with a regular cycle were used in the experiments.

### Experimental groups

Animals (n=48) were randomly divided into six groups (8 animals per group): Positive control (N-acetylcysteine: 150 mg/kg, *po*; Sigma Aldrich Chemical Co.) (NAC+DOX); Negative control, DOX (10 mg/kg) and saline (SAL+DOX); 0.1 mg/kg *Aloe vera* and DOX (AV0.1+DOX); 1.0 mg/kg *Aloe vera* and DOX (AV1.0+DOX); 10.0 mg/kg *Aloe vera* and DOX (AV10.0+DOX); and Control (0.15 M NaCl vehicle, 0.03 mL) (SAL+SAL). The animals in the positive control group were pre-treated with N-acetylcysteine orally (*po*) and, after 1 h, DOX was administered intraperitoneally (*ip*) in a single dose. In the negative control, the mice were pre-treated with saline (0.15 M NaCl, 0.3 mL; *po*) and after 1 h, DOX (*ip*) was administered in a single dose. The other groups of mice were pre-treated with different concentrations (0.1, 1.0, or 10.0 mg/kg; *po*) of *Aloe vera* and then received a single dose of DOX (*ip*) after 1 h. In the control group, the animals were pre-treated with saline (0.15 M NaCl, 0.3 mL; *po*) and after 1 h, with saline (0.15 M NaCl, 0.15 mL; *ip*) in a single dose. Then, *Aloe vera* was administered once a day for 3 days. On the fourth day, i.e., 24 h after the last administration of *Aloe vera* at different concentrations, the ovaries were collected.

### Analysis of ovarian follicles and stromal cell density

The ovaries were fixed in 10% buffered formalin for 24 h, dehydrated in ethanol, clarified with xylene and embedded in paraffin wax. Then, 5-µm sections were mounted on slides and stained with hematoxylin-eosin (HE). The follicles were classified as primordial or growing follicles (primary, secondary, and antral follicles) and as morphologically normal when an intact oocyte was surrounded by well-organized granulosa cells, without pycnotic nucleus. Follicles with retracted oocytes, pycnotic nucleus, and/or surrounded by disorganized granulosa cells were classified as degenerated (8).

To evaluate stromal cell density, the number of cells in 100 μm^2^ was counted and the average number of stromal cells per field was calculated as previously described (22).

### Analysis of collagen fiber in extracellular matrix

To evaluate collagen fibers, ovarian sections were stained with Picrosirius red (Abcam Kit, ab246832, USA) according to Rittié (23). The percent area occupied by collagen fibers in ten different fields in ovaries from each treatment group was measured using a camera attached to a microscope (Nikon, Eclipse, TS 100, Japan). The images were analyzed by ImageJ software (version 1.51p, 2017, NIH, USA) at 400x magnification. The collagen fibers were marked in red, while the follicles remained colorless. The software automatically excludes the circumference of unstained follicles from the total area marked in red. The percent area of collagen fibers in tissues and the staining intensity were determined by measuring the average pixel intensity of the total area after background subtraction (ImageJ Software).

### Immunohistochemistry assay

Ovaries were fixed in 10% buffered formalin, dehydrated with ethanol, clarified in xylene, and embedded in paraffin. Sections (5-μm thick) from each ovary were mounted on Starfrost glass slides (Knittel, Germany) and used for immunohistochemical reaction according to Barberino et al. (24). The slides were incubated in citrate buffer at 95°C in a decloaking chamber (Biocare, USA) for 40 min to retrieve antigenicity, and endogenous peroxidase activity was prevented by incubation with peroxidase blocker (H_2_O_2_) for 10 min. Nonspecific binding sites were blocked using 10% normal goat serum for 10 min. Then, the sections were incubated in a humidified chamber at room temperature with anti-*TNF-α* antibody for 50 min. Subsequently, the sections were incubated for 20 min with EasyLink One (EasyPath, Brazil) polymer, stained with DAB, and counterstained with hematoxylin for 1 min. For negative control, the tissues were incubated with blocking buffer, without the primary antibody. TNF-α immunostaining in the ovarian tissue was classified as absent, weak, moderate, or strong (6).

### Quantification of *SOD*, *CAT*, *TNF-α*, and *NRF2* mRNA levels

The ovaries were stored at -80°C until total RNA extraction. TRIzol^®^ purification kit (Invitrogen, Brazil) was used to perform total RNA extraction, according to the manufacturer's instructions. The qPCR reactions were composed of 1 μL cDNA as a template in 9.4 μL of SYBR Green Master Mix (PE Applied Biosystems, USA), 9.4 μL of ultra-pure water, and 0.5 μM of each primer. The primers were designed to specifically amplify *SOD*, *CAT*, *TNF-α*, *NRF2* mRNAs and glyceraldehyde-3-phosphate dehydrogenase (*GAPDH*), which was used as reference gene (Table 2). Melting curve analysis of PCR products was used to confirm the specificity of each primer pair. The thermal cycling profile for the first round of qPCR was initial denaturation and polymerase activation for 10 min at 95°C, followed by 40 cycles of 15 s at 95°C, 30 s at 58°C, and 30 s at 72°C (Step One Plus instrument, Applied Biosystems). The final extension was performed at 72°C for 10 min. The 2^ΔΔCt^ method was used to transform Ct values into normalized values for relative expression levels (25).

**Table 2:** Primer pairs used for real-time PCR.

Target gene	Primer sequence (5′→3′)	GenBank accession No.
*GAPDH*	F: GAACGGATTTGGCCGTATTG R: GTGAGTGGAGTCATACTGGAAC	GU214026.1
*SOD*	F: CTCGTCTTGCTCTCTCTGGTC R: CTTGCCTTCTGCTCGAAGTG	NM_011434.2
*CAT*	F: CCAATGGCAATTACCCGTCC R: CCTTGTGAGGCCAAACCTTG	NM_009804.2
*TNF-α*	F: CAGAAAGCATGATCCGCGAC R: CCGCCTGGAGTTCTGGAAG	D84199.2
*NRF2*	F: TTGCCCTAGCCTTTTCTCCG R: CTAGGAGATAGCCTGCTCGC	NM_010902.4

### Statistical analysis

The GraphPad Prism (8.0, USA) software was used to perform the statistical analyses. Chi-squared test was used to compare the rate of normal follicles, as well as those of primordial and developing follicles. Data of collagen fiber distribution and stromal cell density were found to be normally distributed and analyzed by analysis of variance (ANOVA) and Tukey test. *SOD*, *CAT*, *TNF-α*, and *NRF2* mRNA levels were compared by Kruskal-Wallis test, followed by Dunn's multiple comparison test. The results are reported as means±SEM. Differences were considered significant when P<0.05.

## Results

### Evaluation of follicular morphology

**Figure 1:**
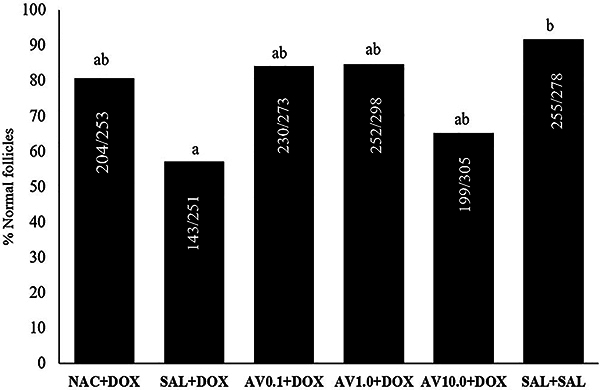
Percentage of normal follicles in ovaries of mice treated with doxorubicin (DOX), saline (SAL), N-acetylcysteine (NAC), and *Aloe vera* (AV) (0.1, 1.0, or 10.0 mg/kg). Different letters indicate significant differences between treatments (P<0.05; ANOVA).

**Figure 2:**
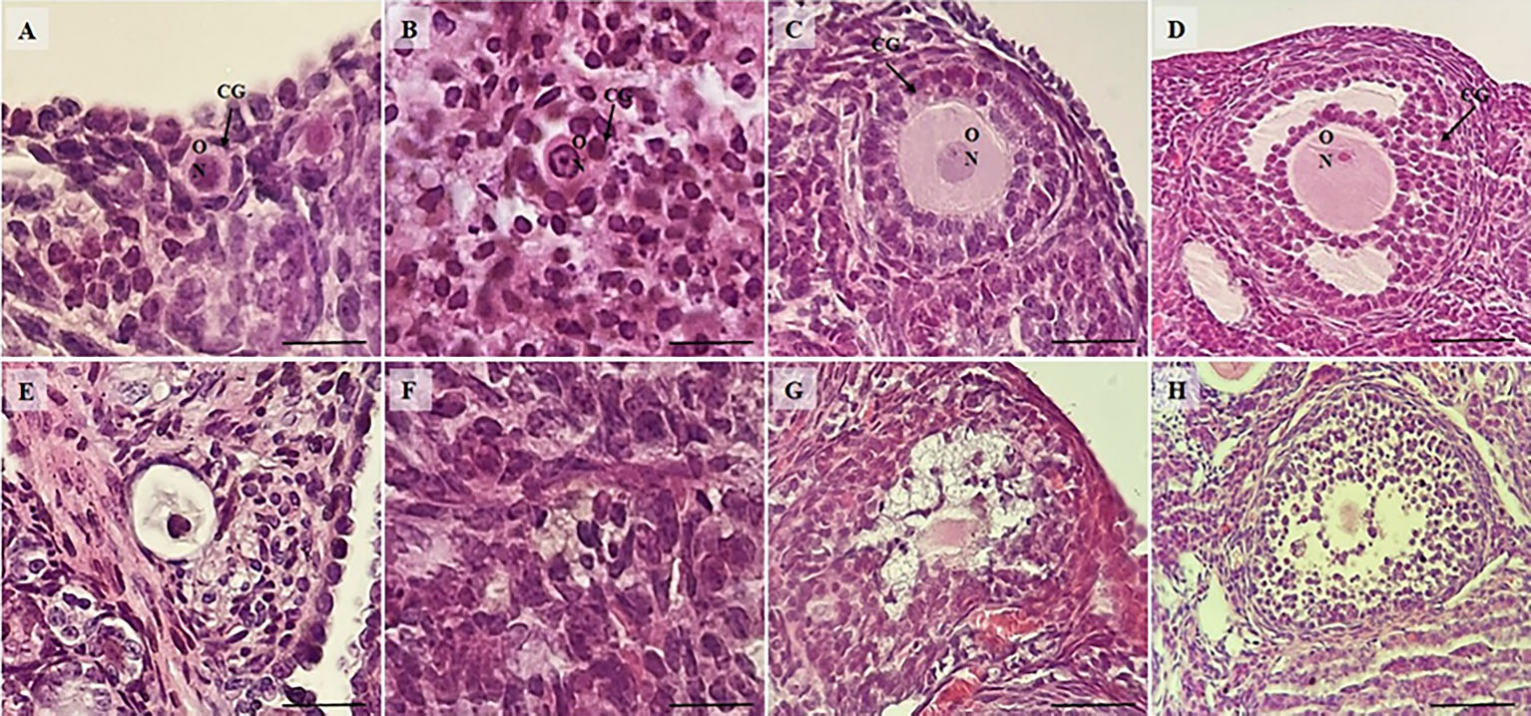
Morphologically normal and degenerated primordial (**A** and **E**), primary (**B** and **F**), secondary (**C** and **G**), and tertiary follicles (**D** and **H**), respectively. GC: granulosa cells; O: oocyte; N: oocyte nucleus. Arrows indicate follicles. Scale bar: 100 μm.

Mice treated with saline solution and DOX had lower percentage of normal follicles compared to those treated only with saline solution. However, animals treated with NAC and DOX or *Aloe vera* (0.1, 1.0 mg/kg) and DOX had normal percentages of follicles similar to those that received saline solution, indicating a protective effects of the substance. No significant differences were found among mice treated with NAC and DOX, *Aloe vera* (0.1, 1.0, or 10.0 mg/kg) and DOX, or saline solution alone (Figure 1). Figure 2A-H shows the morphology of normal or degenerated follicles.

**Figure 3:**
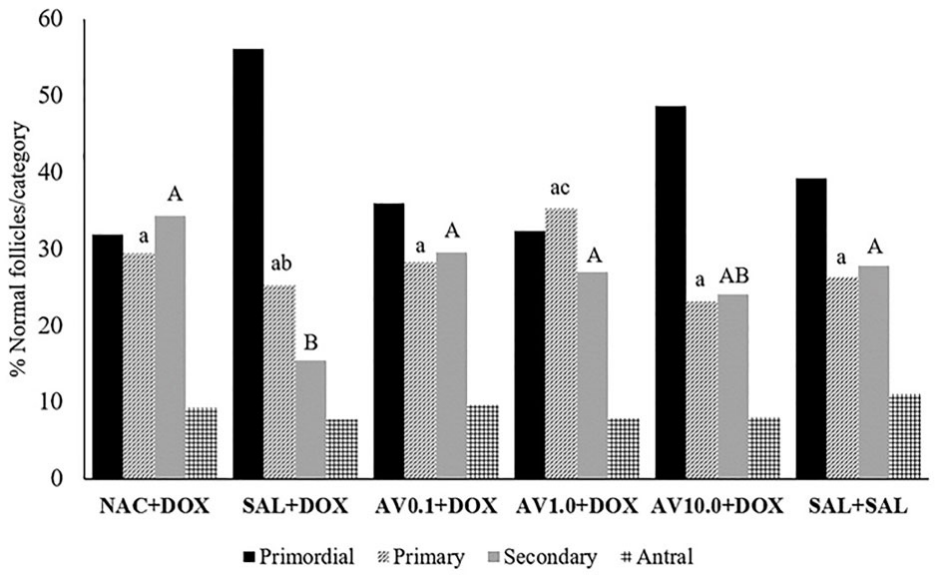
Percentage of normal follicles per category in ovaries of mice treated with doxorubicin (DOX), saline (SAL), N-acetylcysteine (NAC), and *Aloe vera* (AV) (0.1, 1.0, or 10.0 mg/kg). Different letters indicate significant differences between treatments (P<0.05; ANOVA).

Ovaries of mice from all treatment groups had similar percentages of normal primary follicles compared to the saline group. However, animals treated with both 1.0 mg/kg *Aloe vera* and DOX showed a higher percentage of primary follicles than the animals treated with saline solution and DOX. Furthermore, mice treated with NAC and DOX, and 0.1, 1.0, or 10.0 mg/kg *Aloe vera* and DOX showed a similar percentage of normal secondary follicles compared to the animals that received only saline solution. Similarly, mice treated with saline solution and DOX or 10.0 mg/kg *Aloe vera* and DOX showed a similar percentage of normal secondary follicles. The percentages of primordial and antral follicles did not differ among treatment groups (Figure 3).

### Analysis of collagen fibers in the ovaries

**Figure 4:**
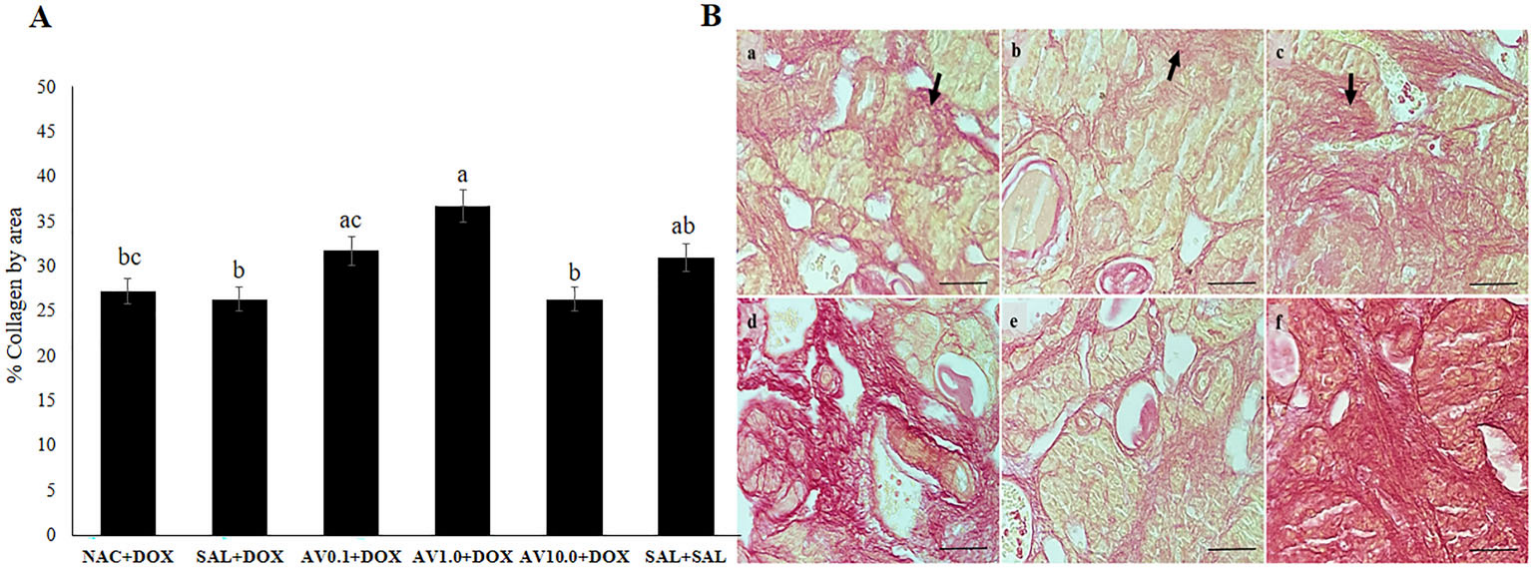
**A**, Collagen levels (means±SEM) in the ovaries of mice treated with doxorubicin (DOX), saline (SAL), N-acetylcysteine (NAC), and *Aloe vera* (AV) (0.1, 1.0, or 10.0 mg/kg). Different letters indicate significant differences between treatments (P<0.05; ANOVA). **B**, Collagen fibers (arrows) labeled by Picrossirius red. **a**, NAC+DOX; **b**, SAL+DOX; **c**, *Aloe vera* 0.1+DOX; **d**, *Aloe vera* 1.0+DOX; **e**, *Aloe vera* 10.0+DOX; **f**, SAL+SAL. Scale bar: 100 μm.

Mice treated with 0.1 or 1.0 mg/kg *Aloe vera* and DOX showed a higher percentage of collagen fibers per area than those treated with saline solution and DOX or 10.0 mg/kg *Aloe vera* and DOX. Mice treated with NAC and DOX, saline solution and DOX, or 10.0 mg/kg *Aloe vera* and DOX showed a similar percentage of collagen fibers per area to the animals treated only with saline solution (Figure 4A). Figure 4B shows collagen fibers in mice ovaries.

### Stromal cell density

**Figure 5:**
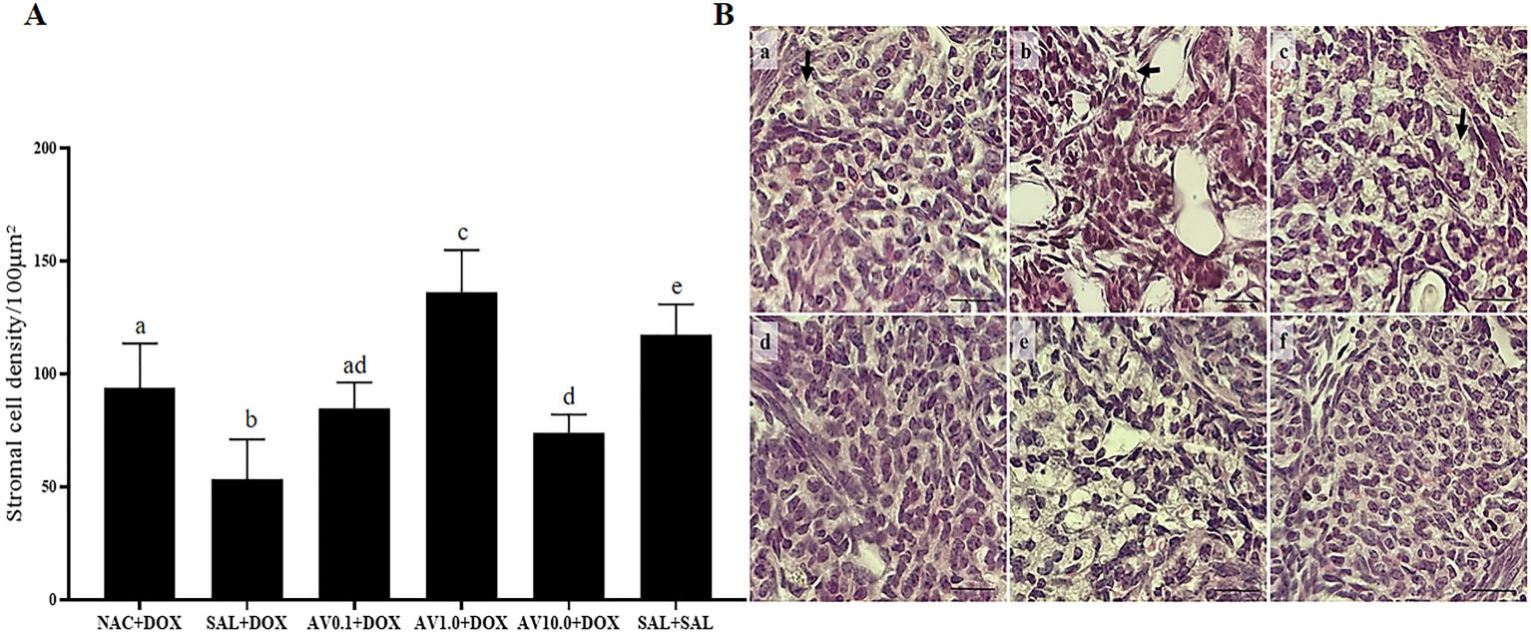
**A**, Stromal cell density (means±SEM) in ovaries of mice treated with doxorubicin (DOX), saline (SAL), N-acetylcysteine (NAC), and *Aloe vera* (AV) (0.1, 1.0, or 10.0 mg/kg). Different letters indicate significant differences between treatments (P<0.05; ANOVA). **B**, Representative images of ovarian stromal cell density in ovaries of mice. **a**, NAC+DOX; **b**, SAL+DOX; **c**, *Aloe vera* 0.1+DOX; **d**, *Aloe vera* 1.0+DOX; **e**, *Aloe vera* 10.0+DOX; **f**, SAL+SAL. Arrows indicate areas with affected stroma. Scale bar: 100 μm.

As shown in Figure 5A, animals treated with 1.0 mg/kg of *Aloe vera* and DOX and those treated with saline alone exhibited a higher density of stromal cells compared to the other treatments. Furthermore, animals treated with NAC and DOX or 0.1 mg/kg *Aloe vera* and DOX showed similar stromal cell densities. Animals treated with 0.1 or 10.0 mg/kg *Aloe vera* together with DOX showed comparable stromal cell densities. On the other hand, the ovaries of animals treated with DOX and saline demonstrated a lower density of stromal cells. Figure 5B illustrates the ovarian stroma of all groups.

### TNF-α immunostaining

**Figure 6:**
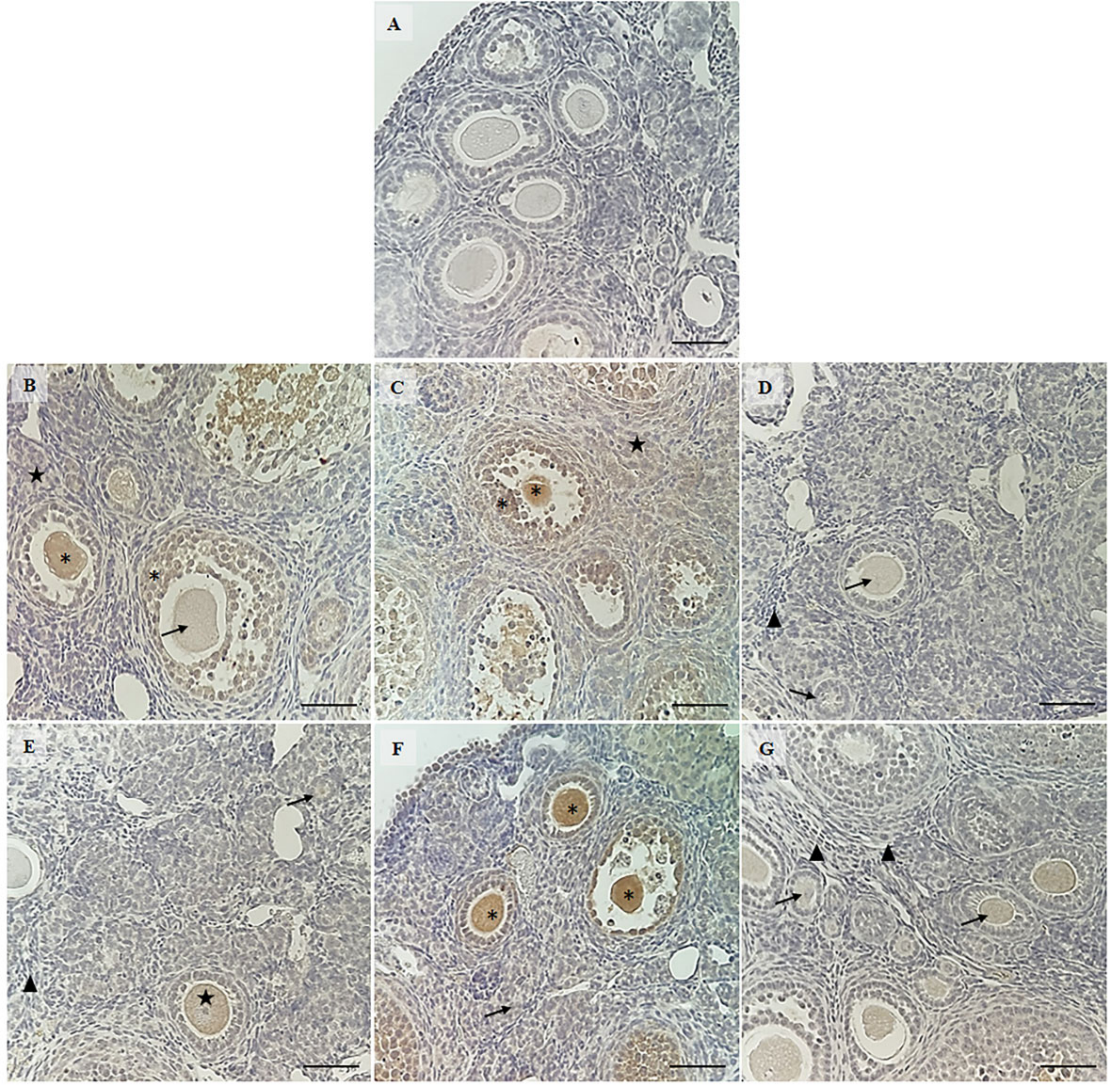
Immunohistochemical staining for TNF-α in ovaries of mice treated with doxorubicin (DOX), saline (SAL), N-acetylcysteine (NAC), *Aloe vera* (AV) (0.1, 1.0, or 10.0 mg/kg), and control groups. **A**, Negative control; **B**, NAC+DOX; **C**, SAL+DOX; **D**, *Aloe vera* 0.1+DOX; **E**, *Aloe vera* 1.0+DOX; **F**, *Aloe vera* 10.0+DOX; **G**, SAL+SAL. Triangles indicate absent coloration; arrows indicate weak coloration, stars indicate moderate coloration; asterisks indicate strong coloration. Scale bar: 100 μm.

The immunohistochemistry results (Figure 6) show that mice treated with saline solution and DOX showed weak labeling for *TNF-α* in the oocyte of primordial follicles, while granulosa cells and oocytes of secondary and antral follicles had strong staining. Moderate staining was seen in the ovarian stroma. Furthermore, animals treated with *Aloe vera* (0.1 and 1.0 mg/kg) and DOX or only saline solution showed absent staining in primordial follicles, weak or moderate staining in primary, secondary, and antral follicles, and absent staining in ovarian stroma. The reaction control showed no staining.

### *TNF-α*, *NRF2*, *CAT*, and *SOD* mRNA levels

**Figure 7:**
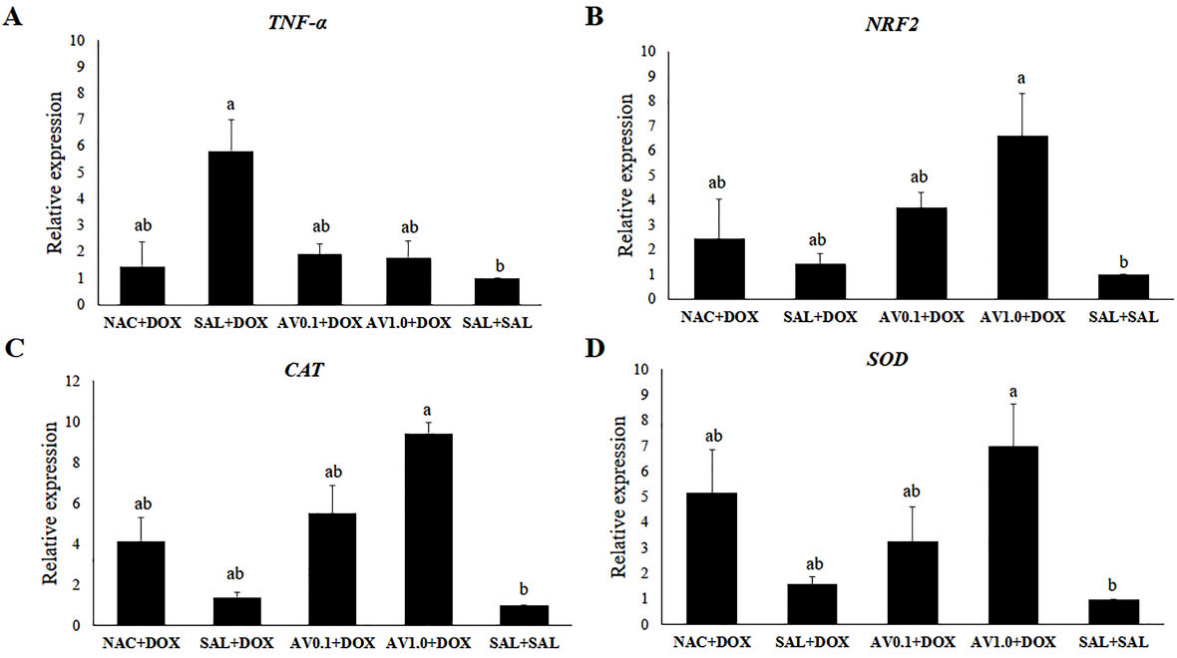
mRNA levels (means±SEM) for (**A**) tumor necrosis factor (TNF)-α, (**B**) nuclear factor erythroid 2-related factor 2 (NRF2), (**C**) superoxide dismutase (SOD), and (**D**) catalase (CAT) in mice treated with doxorubicin (DOX), saline (SAL), N-acetylcysteine (NAC), and *Aloe vera* (0.1 and 1.0 mg/kg). Different letters indicate significant differences between treatments (P<0.05; ANOVA).

Ovaries from mice treated with saline solution and DOX had a higher expression of *TNF-α* than those treated with saline solution only. In addition, animals treated with NAC and DOX or *Aloe vera* (0.1 and 1.0 mg/kg) and DOX showed a similar expression for *TNF-α* to mice treated only with saline solution (Figure 7A). However, mice treated with NAC and DOX, saline solution and DOX, or *Aloe vera* (0.1 and 1.0 mg/kg) and DOX showed no difference in *TNF-α* mRNA levels. Furthermore, the animals treated with 1.0 mg/kg *Aloe vera* and DOX showed a higher expression for *NRF2*, *CAT*, and *SOD* than those treated only with saline solution. However, the mice treated with NAC and DOX, saline solution and DOX or 0.1 mg/kg *Aloe vera* and DOX, and only saline solution showed no difference in *NRF2*, *CAT,* and *SOD* levels (Figure 7B-D).

## Discussion

This study found that co-administration of *Aloe vera* extract reduced DOX-induced damage in mice ovaries. This protective effect was characterized by a higher proportion of normal follicles, preservation of the ovarian stroma, and an increase in mRNA levels of antioxidant enzymes. These findings strongly suggest that *Aloe vera* extract protects the ovaries by enhancing the antioxidant response, thereby mitigating the severe damage inflicted by DOX. The identification of protective agents capable of preventing ovarian damage during antitumor treatment represents a fundamental advance in the preservation of fertility in cancer patients (26). The *Aloe vera* extract (0.1, 1.0, or 10.0 mg/kg) maintained the percentage of normal follicles similar to the control group and protected the ovarian stroma against DOX toxic effects.

DOX treatment reduced stromal density and consequently affected the supply of nutrients required for follicular development (26). Previous studies also showed that DOX damaged ovarian microvasculature and increased ROS production, which triggers apoptosis in stromal cells (2). It has been reported that *Aloe vera* can induce angiogenesis (27). Thus, we believe the antioxidant and angiogenic activities of *Aloe vera* extract reduced ROS production and protected the ovarian vascular network, protecting stromal cells and follicles against DOX toxicity (28). It is important to highlight that DOX administration results in vascular damage evidenced by reduced ovarian blood flow and decreased ovarian size (29). Primordial and early-preantral follicles do not have their own individual vascular supply and depend on blood vessels in the surrounding stroma (30).

In our study, ovaries of mice treated with *Aloe vera* extract (1.0 mg/kg) showed a higher percentage of collagen fibers. The maintenance of collagen and ovarian stromal cells observed in the *Aloe vera*-treated mice may have contributed to the maintenance of the percentage of morphologically normal follicles, since the extracellular matrix (ECM) is composed of a protein network rich in collagen, polysaccharides and water. The stabilization of the ECM structure also allows a specialized microenvironment to regulate cell activity and development, including cell proliferation and survival, steroidogenesis, regulation of cell aggregation, and morphology (17). During inflammatory conditions or tissue damage, the expression of matrix metalloproteinases (MMP) is increased by the action of pro-inflammatory cytokines, such as TNF-α and transforming growth factor beta (TGF-β) (31). MMPs have the function of degrading ECM macromolecules, including collagen, fibronectin, laminin, and proteoglycans (32). Sánchez et al. (33) showed that *Aloe vera* gel had a protective effect in mice with alcohol-induced gastritis by increasing the inhibitory activity of MMP-9. Furthermore, Yagi (34) showed the ability of aloe emodin, a compound from *Aloe vera*, to reduce the cytotoxicity of TNF-α.

In the present study, DOX increased TNF-α protein expression in secondary and antral follicles, as well as in ovarian stroma. In contrast, animals treated with both *Aloe vera* (0.1 and 1.0 mg/kg) and DOX showed no immunostaining in primordial follicles and ovarian stroma, whereas primary, secondary, and antral follicles showed weak or moderate immunostaining. The cyclooxygenase (prostaglandin catalyst) inhibition may contribute to the anti-inflammatory and antioxidant properties of *Aloe vera* (35). The phytosterols (campesterol, β-sitosterol, lupeol, and cholesterol) present in *Aloe vera* have been shown to potentiate the anti-inflammatory actions (36). Nguyen et al. (37), using molecular docking, showed that many components of *Aloe vera* can directly interact with TNF-α, suggesting that binding to active compounds can destabilize TNF-α, shorten its half-life, or suppress its production since TNF-α can trigger apoptosis through activation of caspase-8. In this study, we observed that animals treated with DOX exhibited elevated *TNF-α* expression levels. However, when *Aloe vera* was administered at doses of 0.1 and 1.0 mg/kg, *TNF-α* expression levels resembled those of the animals in the control group. In a previous study, it was observed that *Aloe vera* can elevate the level of interleukin-10 (IL-10), an anti-inflammatory cytokine produced by various cells, including monocytes/macrophages and T lymphocytes (28). Therefore, *Aloe vera* showed beneficial effects in reducing inflammation, being able to neutralize the TNF-α present in the tissue and reduce its expression.

Our results also demonstrated that *Aloe vera* extract (1.0 mg/kg) can elevate the expression of *NRF2* and antioxidant enzymes such as *SOD* and *CAT*, thereby contributing to the preservation of the antioxidant environment and safeguarding against damage induced by DOX. Oxidative stress is one of the primary mechanisms responsible for DOX toxicity (6). The structure of DOX has quinone and hydroquinone portions that form semiquinone radical intermediates, leading to increased oxidative stress and depletion of antioxidant defenses (38). Enzymes of the antioxidant system are actively involved in the maintenance of oxidant/antioxidant homeostasis and in the detoxification of xenobiotics in the biological system (36). Previous studies show that *Aloe vera* extract could increase the activity of important antioxidant enzymes such as CAT and SOD and increase GSH in plasma and tissues (39). In addition, glucomannan from *Aloe vera* extract activated NRF2, decreased ROS levels, and maintained the integrity of the intestinal barrier in mice (40).

In conclusion, co-administration of *Aloe vera* extract in DOX-treated mice helped preserve ovarian follicles and stromal cells. The *Aloe vera* extract reduced ROS through antioxidant enzymes and inhibited *TNF-α* expression. These findings suggest that *Aloe vera* extract may have potential as an adjuvant therapy in clinical settings, offering a protective strategy for patients undergoing chemotherapy, particularly in preserving ovarian function and fertility. Further clinical studies are needed to explore its efficacy and safety in humans.
